# Management of Flow Reversal in Patients With Atrial Shunts Implanted to Reduce Left Atrial Pressure

**DOI:** 10.1016/j.jaccas.2025.103893

**Published:** 2025-07-03

**Authors:** Hidenori Yaku, Jean-Christophe Eicher, Martin W. Bergmann, Tobias Kister, Tudor C. Pörner, Barry A. Borlaug, Rajeev C. Mohan, Matthew Price, Jan Komtebedde, Sanjiv J. Shah

**Affiliations:** a Division of Cardiology, Northwestern University Feinberg School of Medicine, Chicago, Illinois, USA; b Department of Cardiology, François Mitterrand University Hospital, Dijon, France; c Department of Cardiology and Intensive Care Medicine, Asklepios Klinik Altona, Hamburg, Germany; d Department of Cardiology, Heart Center Leipzig at University of Leipzig, Leipzig, Germany; e Division of Cardiology, Angiology, Pneumology and Intensive Medical Care, Department of Internal Medicine, University Hospital Jena, Friedrich Schiller University Jena, Jena, Germany; f Department of Cardiovascular Medicine, Mayo Clinic, Rochester, Minnesota, USA; g Scripps Clinic, La Jolla, California, USA; h Corvia Medical, Inc, Tewksbury, Massachusetts, USA

**Keywords:** heart failure with preserved ejection fraction, hemodynamics, interventional cardiology, right heart catheterization, treatment

## Abstract

This case series presents 6 patients with heart failure (HF) and preserved or mildly reduced ejection fraction who underwent atrial shunt implantation in a clinical trial evaluating its safety and efficacy. Shunt closure was considered in selected cases of worsening HF, particularly when shunt flow reversal was observed. Detailed hemodynamic evaluation revealed that shunt closure may have been detrimental in 5 cases and beneficial in only 1 case. Intentional closure of an implanted atrial shunt has not been previously reported; here, we describe the clinical decision-making process and outcomes based on comprehensive hemodynamic assessments, including pulmonary vascular dynamics and shunt flow patterns in such cases. These findings highlight the critical importance of thorough hemodynamic evaluation in patient management after atrial shunt placement in recognition of other factors that may play a role in the presence of the shunt. Additional research is needed to establish optimal management strategies for patients experiencing worsening HF after atrial shunt implantation.

## Graphical Abstract

**Figure:**
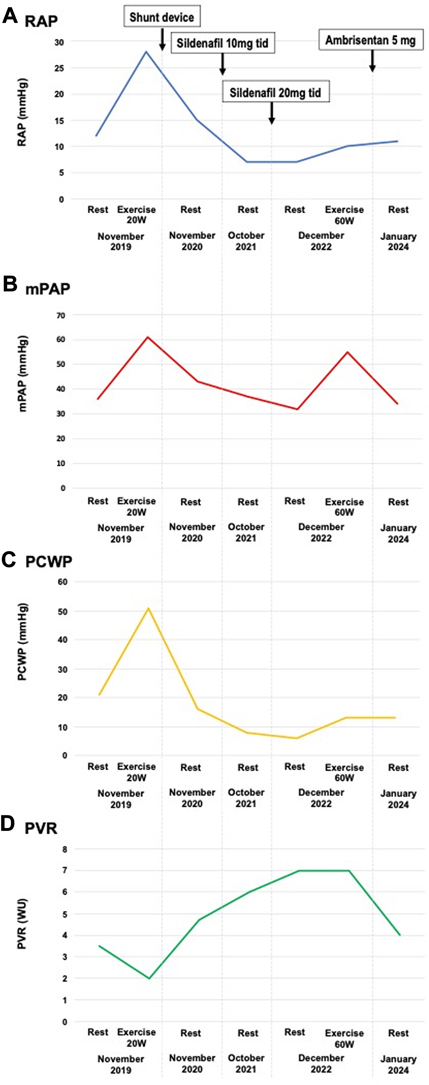

Atrial shunts have been studied in clinical trials to treat patients with heart failure (HF).^1^, ^2^, ^3^ The goal is to decrease left atrial pressure (LAP) at rest and during exertion. If HF symptoms worsen after shunt implantation, closure may be considered, particularly when bidirectional shunt flow or flow reversal is present. To date, there have been no reports of intentional closure of an atrial shunt after implantation.

**Take-Home Messages**

• In patients with worsening HF after atrial shunt implantation, shunt closure may inadvertently worsen LA hypertension and should not be considered the default solution.
• Comprehensive invasive hemodynamic evaluation, including temporary balloon occlusion testing, is essential before considering shunt closure in patients with worsening symptoms.
• Alternative interventions, such as transcatheter tricuspid valve repair or pulmonary vasodilator therapy, may be more appropriate than shunt closure for selected patients based on individualized hemodynamic evaluation.

Whether shunt closure is appropriate depends on various factors, including the direction of flow, right ventricular (RV) function, tricuspid regurgitation (TR), and pulmonary vascular hemodynamics. Herein, we present a case series of 6 patients with worsening HF after atrial shunt implantation.

## Case Series

Table 1, Table 2, Table 3, Table 4, Table 5, Table 6 summarize the key findings of 6 patients. In 5 patients, after careful diagnostic evaluation, shunt closure was deemed to likely be detrimental. In 1 patient, it was deemed appropriate and successfully performed in combination with tricuspid valve repair.

**Table 1:** Case 1: Invasive Hemodynamic Data

Date of Measurement	November 2019		November 2020	October 2021	December 2022		January 2024
	Baseline (Preimplant)			After Starting Sildenafil			After Adding Ambrisentan
	Rest	Exercise 20 W	Rest	Rest	Rest	Exercise 60 W	Rest
RAP, mm Hg	12	28	15	7	7	10	11
mPAP, mm Hg	36	61	43	37	32	55	34
PCWP, mm Hg	21	51	16	8	6	13	13
Cardiac output, L/min	4.2	5.1	5.7	4.8	3.7	6	5.2
Cardiac index, L/min/m^2^	2.4	2.9	3.4	2.9	2.3	3.7	3.2
PVR WU	3.5	2	4.7	6	7	7	4

mPAP = mean pulmonary artery pressure; PCWP = pulmonary capillary wedge pressure; PVR = pulmonary vascular resistance; RAP = right atrial pressure.

**Table 2:** Case 2: Invasive Hemodynamic Data

Date of Measurement	October 2019		May 2022			
	Baseline (Preimplant)					
	Rest	Exercise	Rest	Exercise (Handgrip)	Occlusion (Balloon)	Exercise + Occlusion
RAP, mm Hg	12	19	9	NA	NA	NA
mPAP, mm Hg	28	41	28	NA	NA	NA
PCWP, mm Hg	18	32	16^a^	27^a^	24^a^	34^a^
V-wave, mm Hg	31	55	25^a^	39^a^	38^a^	50^a^
Cardiac output, L/min	NA	NA	6.9	NA	NA	NA
Cardiac index, L/min/m^2^	NA	NA	3.2	NA	NA	NA
PVR, WU	1.5	NA	2.2	NA	NA	NA

LAP = left atrial pressure; NA = not applicable; other abbreviations as in Table 1.

a Direct LAP measurement.

**Table 3:** Case 3: Invasive Hemodynamic Data

Date of Measurement	2018		2022	
	Baseline (Preimplant)			
	Rest	Exercise	Rest	Ballon Occlusion
RAP, mm Hg	21	20	20	19
mPAP, mm Hg	58	56	41	48
PCWP, mm Hg	15	15	21	18
Cardiac output, L/min	5.8	5.9	8.1	7.9
Cardiac index, L/min/m^2^	NA	NA	NA	NA
PVR, WU	NA	NA	2.5	3.8
Q_p_/Q_s_	NA	NA	1.2^a^	NA

Q_p_/Q_s_ = pulmonary/systemic flow ratio; other abbreviations as in Tables 1 and 2.

a Left-to-right flow.

**Table 4:** Case 4: Invasive Hemodynamic Data

Date of Measurement	April 2018		February 2022		December 2022			
	Baseline (Preimplant)							
	Rest	Exercise 80 W	Rest	Exercise 40 W	Rest	Legs Raised	Exercise 20 W	Exercise 80 W
RAP, mm Hg	7	15	17	20	9	13	18	18
mPAP, mm Hg	26	46	30	50	32	NA	NA	48
PCWP, mm Hg	16	33	20	25	18	25	31	31
V-wave, mm Hg	NA	NA	NA	NA	29	37	37	43
Cardiac output, L/min	3.9	6.3	3.1	4.8	3.3	NA	NA	7.8
Cardiac index, L/min/m^2^	2.2	3.6	1.8	2.7	1.9	NA	NA	4.5
PVR, WU	2.3	2	2.7	5.3	4.2	NA	NA	2.6

Abbreviations as in Table 1.

**Table 5:** Case 5: Invasive Hemodynamic Data

Date of Measurement	July 2020			September 2024		
	Baseline (Preimplant)			Rest Only		
	Rest	Exercise 20 W	Exercise 40 W	Pre-TEER	Post Balloon Occlusion	Post-TEER
RAP, mm Hg	8	8	20	12	NA	8
mPAP, mm Hg	41	41	48	30	40	NA
LVEDP, mm Hg	NA	NA	NA	NA	NA	NA
PCWP, mm Hg	29	35	43	17	29	NA
V-wave, mm Hg	43	48	48	31	49	NA
Cardiac output, L/min	4.97	NA	5.7	NA	NA	NA
Cardiac index, L/min/m^2^	2.61	NA	3	NA	NA	NA
PVR, WU	2.4	NA	0.9	NA	NA	NA

LVEDP = left ventricular end-diastolic pressure; TEER = transcatheter edge-to-edge repair; other abbreviations as in Tables 1 and 2.

**Table 6:** Case 6: Invasive Hemodynamic Data

Date of Measurement	November 2019		May 2023		
	Baseline (Preimplant)		Prior to Shunt Occlusion		Post Shunt Occlusion
	Rest	Exercise 40 W	Rest	Ballon Occlusion	Rest
RAP, mm Hg	1	1	19	16	14
mPAP, mm Hg	20	35	28	30	26
PCWP, mm Hg	15	32	15	15	14
V-wave, mm Hg	20	39	25	24	24
Cardiac output, L/min	3	3.4	2.91	2.8	2.71
Cardiac index, L/min/m^2^	1.65	1.87	1.6	1.6	1.53
PVR, WU	1.7	3.8	3.5	3.4	3.5

Abbreviations as in Table 1.

## Case 1

**Figure 1:**
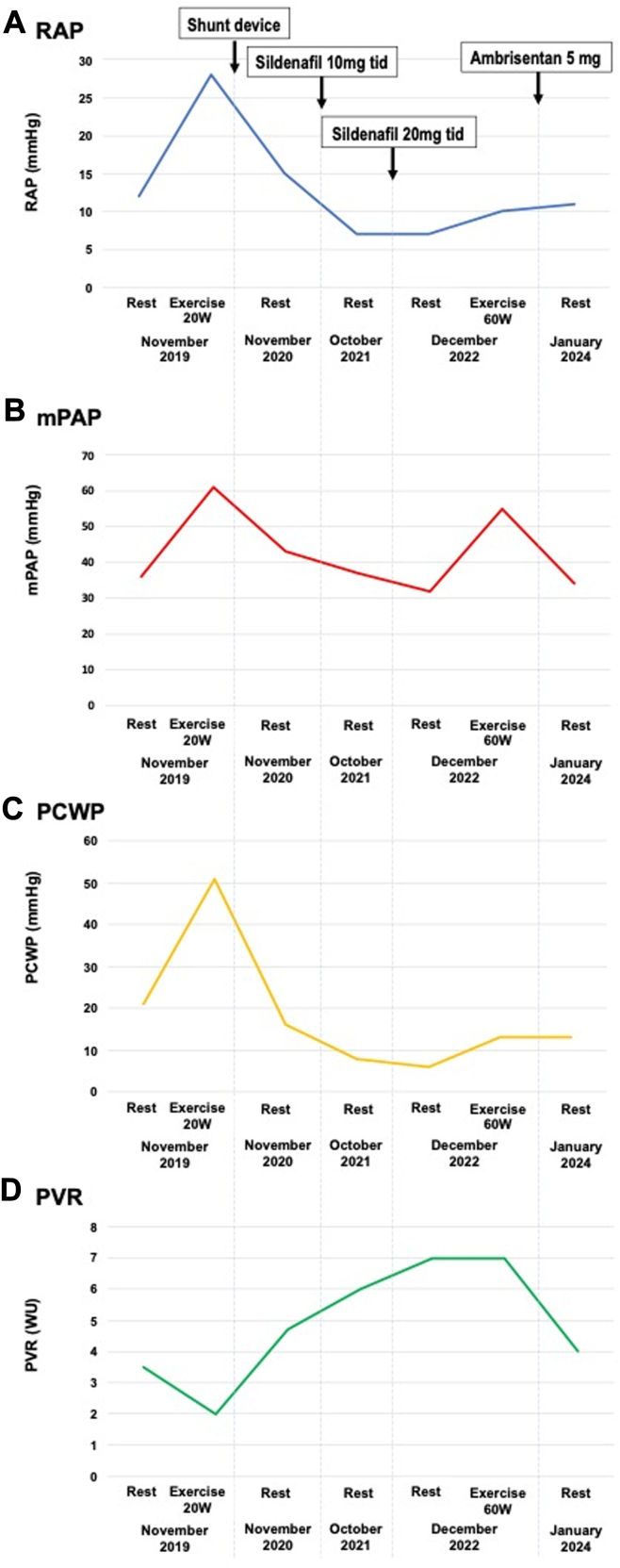
Time Course of Exercise Invasive Hemodynamics (A) Right atrial pressure (RAP); (B) mean pulmonary artery pressure (mPAP); (C) pulmonary capillary wedge pressure (PCWP); and (D) pulmonary vascular resistance (PVR).

An 83-year-old female patient with hypertension and paroxysmal atrial fibrillation (AF) presented with exercise-induced dyspnea and leg edema 5 years prior. She was diagnosed with heart failure with preserved ejection fraction (HFpEF) and enrolled in the REDUCE LAP-HF II (A Study to Evaluate the Corvia Medical, Inc IASD System II to Reduce Elevated Left Atrial Pressure in Patients with Heart Failure) trial, studying the Corvia Atrial Shunt (8-mm diameter).^2^ Initial transthoracic echocardiography (TTE) displayed normal left ventricular volume, LA enlargement (38 mL/m^2^), moderate RV dilatation, and estimated systolic pulmonary artery pressure (PAP) of 55 mm Hg. N-terminal pro–B-type natriuretic peptide (NT-proBNP) was elevated (723 pg/mL). Right heart catheterization (RHC) required by the trial protocol revealed combined pre- and postcapillary pulmonary hypertension (PH) (Figure 1, Table 1). Exercise induced a dramatic increase in pulmonary capillary wedge pressure (PCWP) from 21 to 51 mm Hg and a decrease in pulmonary vascular resistance (PVR) from 3.5 to 2 WU at 20 W, resulting in transient acute pulmonary edema that required treatment with intravenous furosemide. The patient was randomized to the treatment arm, and a shunt was implanted without complication.

**Figure 2:**
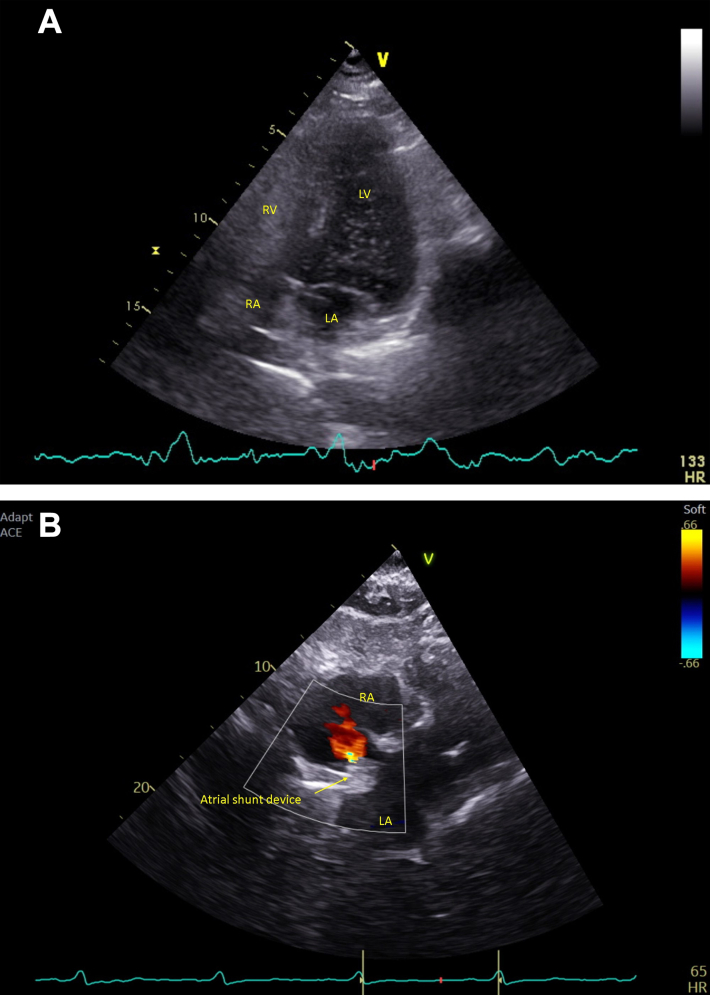
Echocardiographic Findings of Sequential Changes in Shunt Direction (A) Apical 4-chamber view. Right-to-left shunting demonstrated by bubble contrast echocardiography. Bubble contrast echocardiography performed in November 2020 showed right-to-left shunting through interatrial communication. The contrast bubbles appear in the left heart chambers shortly after their injection in the right atrium, confirming the presence of right-to-left shunting. (B) Subcostal view. Left-to-right shunting demonstrated by color flow Doppler echocardiography. Color Doppler echocardiography performed in September 2024 demonstrated left-to-right shunting at the atrial level. The color Doppler signal showed the flow from the left to right atrium, indicating a reversal of the previous right-to-left shunt. Oxygen saturation measurements were normal at this time. LA = left atrium; LV = left ventricle; RA = right atrium; RV = right ventricle.

Following shunt implantation, NT-proBNP decreased slightly to 509 pg/mL but clinically she deteriorated to NYHA functional class IV at 1 year, with right-to-left shunting, systemic desaturation (O_2_ saturation: 88%), and increased NT-proBNP (599 pg/mL) (Figure 2). Repeat RHC demonstrated improved resting PCWP and cardiac output (CO) but increased mean PAP and PVR (Table 1). Further work-up revealed limited cutaneous systemic sclerosis without lung fibrosis, with a 10-year history of worsening Raynaud phenomenon.

Treatment with pulmonary vasodilator therapy was prioritized over shunt closure, because right-to-left shunting was considered a compensatory mechanism in systemic sclerosis–associated pulmonary vasculopathy. She was initiated on furosemide and sildenafil 10 mg 3× a day. She reported significant symptomatic improvement, and RHC at 6 months showed normalization of PCWP and improvement in mean PAP to 37 mm Hg. Sildenafil was then uptitrated to 20 mg 3× a day. One year after dose escalation, PCWP remained normal, and mean PAP had further decreased to 32 mm Hg, with concurrent improvement in exercise tolerance. However, repeat exercise testing showed that mean PAP increased to 55 mm Hg and PVR to 7 WU (Figure 1, Table 1). Ambrisentan 5 mg was added to the treatment and has been well tolerated. The last measured PVR was 4 WU, and she continues to do well 5 years postprocedure.

## Case 2

A 78-year-old male patient with persistent AF (prior pulmonary vein isolation and isthmus ablation) and hypertension presented 7 years prior with NYHA functional class III dyspnea and was diagnosed with HFpEF. TTE revealed PH (systolic PAP: 55 mm Hg) and mild TR. Cardiac magnetic resonance imaging revealed hypertensive heart disease with mild pulmonary vein stenosis. NT-proBNP was 583 pg/mL and RHC showed isolated postcapillary PH (Table 2). He was enrolled in REDUCE LAP-HF II and received a shunt.

At a 6-month follow-up, he improved to NYHA functional class II (NT-proBNP: 723 pg/mL). However, at a 2-year follow-up, he worsened to NYHA functional class III with leg edema, elevated NT-proBNP (3,412 pg/mL), right-to-left shunting, and severe TR. Repeat RHC was performed to determine whether shunt closure was indicated. Balloon occlusion during handgrip exercise increased PCWP and V-wave pressures (Table 2). Simultaneous TTE showed no change in TR severity.

Based on these findings, instead of shunt closure, tricuspid transcatheter edge-to-edge repair (T-TEER) was performed, with only trace residual TR, sustained improvement to NYHA functional class II, and a decrease in NT-proBNP to 1,421 pg/mL at the last follow-up visit, which was approximately 22 months after T-TEER.

## Case 3

A 58-year-old male patient with HFpEF, mild PH, and mild TR received a shunt 6 years prior as part of REDUCE LAP-HF II. Comorbidities included AF, revascularized coronary disease, stage 3 chronic kidney disease, chronic obstructive pulmonary disease, obstructive sleep apnea, and obesity (body mass index: 38.5 kg/m^2^). Baseline hemodynamics are shown in Table 3.

Four years later, he presented with acute decompensated HF (NYHA functional class IV). TTE and transesophageal echocardiography showed an EF of 51%, RV dilation with flattening of the interventricular septum, moderate TR, peak systolic RV pressure of 50 mm Hg, and bidirectional (predominantly right-to-left) shunting. RHC at rest and after a 15-minute balloon occlusion of the atrial shunt resulted in increased PAP (Table 3). Based on the findings, systemic volume overload was thought to be the cause of the patient’s decompensation.

The shunt was not closed, and the volume overload was managed with ultrafiltration and diuretics. He was eventually discharged in improved condition on chronic hemodialysis in September 2022.

## Case 4

A 39-year-old female patient with a history of aplastic anemia (status post radiation and bone marrow transplant), left bundle branch block, and hypertension presented with worsening exertional dyspnea and chest discomfort. She had been very active historically but developed dyspnea over 3 years and was diagnosed with PH (RV systolic pressure: 63 mm Hg) by TTE. Historical catheterization showed resting mean PAP of 42 mm Hg with left ventricular end-diastolic pressure of 17 mm Hg and PCWP of 25 mm Hg with a large V-wave. CO was 4.1 L/min with a PVR of 4.1 WU. Computed tomographic imaging was reportedly negative for pulmonary parenchymal disease and pulmonary veno-occlusive disease. Transesophageal echocardiography showed mitral annular calcification, calcified LA, and possible patent foramen ovale. Repeated catheterization 6 years ago revealed elevated left-sided filling pressures with a prominent V-wave at rest, markedly increasing during exercise (Table 4). The marked elevation in PCWP during exercise was disproportionate to the rise in left ventricular end-diastolic pressure (16 mm Hg), leading to a diagnosis of HFpEF due to stiff LA syndrome. She was treated with atrial septostomy, followed by Occlutech AFR shunt device placement 6 months later on compassionate use approval, with improvement in symptoms to NYHA functional class II.

Three years after shunt implantation, her symptoms again worsened. Hemodynamics showed lower PCWP but worsening PH with elevated exercise PVR and reduced CO. An sodium-glucose cotransporter 2 inhibitor was initiated, and she was maintained on furosemide. Simultaneous echocardiography with agitated saline and RHC showed mild bidirectional shunting, which was balanced and not associated with hypoxemia. At rest, she had mildly elevated biventricular filling pressures, mild to moderately elevated PAPs, and low CO (Table 4). RHC during leg raise showed increased biventricular filling pressures. At 20 W exercise, biventricular filling pressures increased further, and at peak exercise (80 W), biventricular filling pressures were comparable to early exercise, and PAPs were increased (Table 4). Given the absence of hypoxemia and persistent elevation in PCWP, the decision was made not to attempt closure of her device, and she continued to be managed medically with sodium-glucose cotransporter 2 inhibitor and furosemide.

## Case 5

An 89-year-old female patient with HF with mildly reduced EF, AF, and mild TR was enrolled in REDUCE-LAP II 4 years prior due to exertional dyspnea and received a shunt followed by symptomatic improvement. Due to ongoing symptomatic AF, she underwent an atrioventricular node ablation and pacemaker placement 2 years later. Subsequently, she presented with worsening dyspnea and severe TR thought to be due to tricuspid leaflet impingement due to pacemaker leads. The TR had been graded as moderate prior to the permanent pacemaker implant. The RV lead was extracted, and a leadless device was implanted, but the severe TR persisted. Thus, a plan was made to consider shunt closure and possible T-TEER. First, test occlusion was performed while directly measuring LAP, which showed a substantial increase in LAP and V-wave pressures (Table 5). Consequently, the shunt was not closed, and successful T-TEER was performed with mild residual TR 1 year ago. At 1-month follow-up, she demonstrated substantial clinical improvement, with an increase in Kansas City Cardiomyopathy Questionnaire score from 53 to 97 and 6-minute walk distance from 560 to 760 m.

## Case 6

A 78-year-old female patient with HFpEF (NT-proBNP: 858 pg/mL), hypertension, and a history of coronary artery bypass grafting underwent shunt implantation as part of REDUCE LAP-HF II 5 years prior. Preprocedural RHC showed resting mean PAP of 20 mm Hg with PVR of 1.7 WU and peak exercise mean PAP of 35 mm Hg with PVR of 3.8 WU (Table 6). Between 2020 and 2022, serial echocardiography showed progressive RV dilation and worsening TR. The patient was hospitalized for HF with RV dilation and massive TR 3 years after shunt implantation. The decision was made to close the shunt following repeated RHC and perform a T-TEER procedure in the same setting.

During the intervention, hemodynamic assessment with balloon occlusion of the shunt showed decreased percentage of O_2_ saturation in the right atrium and no change in PCWP and systemic or pulmonary hemodynamics. The shunt was subsequently closed using an 18-mm Amplatzer Cribiform Occluder (Abbott Cardiovascular), followed by T-TEER using the PASCAL ACE system (Edwards Lifesciences) as massive TR persisted following shunt closure. Follow-up TTE showed reduced RV dilation and trace TR, and the patient improved clinically.

## Discussion

This case series highlights the complexities of managing patients with HF after atrial shunt implantation. Our experience underscores the importance of comprehensive hemodynamic and imaging evaluation to guide clinical decision-making when patients experience worsening HF symptoms after atrial shunt implantation. Unlike the management of congenital atrial septal defects, where closure decisions primarily depend on shunt size as well as the degree of RV volume and pressure overload, the management of atrial shunt requires careful consideration of left-sided filling pressures in older patients with HFpEF. A prior case report published in 2001 demonstrates the potential deleterious impact of closing a congenital atrial septal defect in an older patient with HFpEF, where doing so unmasked restrictive physiology.^4^ In our series, whereas most patients developed right-to-left shunting and clinical deterioration due to evolving RV dysfunction, pulmonary vascular changes, or worsening TR, shunt closure was only ultimately indicated in 1 patient who benefitted from shunt closure. Importantly, our findings demonstrate that the decision of shunt closure must be individualized, considering the dynamic interplay between LA unloading, RV function, TR severity, and pulmonary hemodynamics, even with right-to-left shunting on echocardiography.

In 5 of the 6 patients, closure was ultimately deemed inappropriate. Each of these cases exemplifies how underlying comorbidities and evolving right-sided structural/functional abnormalities can produce complex hemodynamics following shunt implantation as part of natural disease progression. In case 1, right-to-left shunting initially appeared pathologic, but further evaluation uncovered subclinical systemic sclerosis–associated pulmonary vasculopathy. Thus, pulmonary vasodilators, rather than shunt closure, provided sustained clinical improvement.

Notably, a recurring theme in several of these cases is worsening of TR after atrial shunt implantation. Worsening TR can occur due to tricuspid annular dilation (due to increase in RV and right atrial size from left-to-right shunting through the atrial shunt); RV dysfunction due to pulmonary vascular disease (due to progression of HFpEF or independent causes of pulmonary vascular disease such as autoimmune diseases); primary tricuspid valve pathology (independent of the atrial shunt) such as displacement of pacemaker leads resulting in tricuspid leaflet impingement (case 5). Shunt closure in this context would reduce the volume load on the RV; however, if LAP remains elevated at rest or during exercise, shunt closure could precipitate an increase in LAP and pulmonary congestion, which could impose increased afterload on the RV. The structural interventions on the tricuspid valve proved beneficial in cases 2 and 5, reaffirming that addressing the lesion causing the problem directly is the appropriate management, rather than shunt closure, which may be the initial inclination.

In contrast, case 6 exemplifies a scenario where shunt closure in combination with the structural intervention was justified and effective. The key factor was the demonstration that PCWP and systemic or pulmonary hemodynamics were not adversely altered during temporary balloon occlusion of the shunt. Additionally, severe TR had persisted despite medical optimization. Closing the shunt followed by T-TEER during the same procedure reduced the RV volume overload and resulted in the desired clinical improvement. A temporary test occlusion with no significant increase in LA or pulmonary pressures acutely suggests that shunt closure can be tolerated.

A combination of prespecified and systematic post hoc analyses in REDUCE LAP-HF II identified 2 characteristics that differentiated responders (those who showed a beneficial treatment response) from nonresponders: peak exercise PVR <1.74 WU and absence of cardiac rhythm management devices at baseline.^5^^,^^6^ In retrospect, several patients had characteristics of the nonresponder population, which made them suboptimal candidates for atrial shunting due to pre-existing pulmonary vascular disease.

## Conclusions

In patients with worsening symptoms following atrial shunt implantation, comprehensive hemodynamic evaluation is mandatory for clinical decision-making. Whereas closing the shunt may appear to be a logical solution, it may not be beneficial and could potentially be harmful by inducing further LA hypertension.

## Funding Support and Author Disclosures

Dr Eicher has received consulting fees from Alnylam, Amgen, AstraZeneca, Bayer, Boehringer, Corvia, Novartis, Pfizer, and Vifor. Dr Borlaug has received research grants from AstraZeneca, Medtronic, GlaxoSmithKline, Mesoblast, Novartis, and Tenax Therapeutics; and consulting fees from Actelion, Amgen, Aria, Axon Therapies, Boehringer Ingelheim, Edwards Lifesciences, Eli Lilly, Imbria, Janssen, Merck, Novo Nordisk, and VADovations. Dr Mohan has received consulting fees from Corvia. Dr Komtebedde is employed by Corvia. Dr Shah has received research funding from AstraZeneca, Corvia, and Pfizer; and has received consulting fees from Abbott, Alleviant, Amgen, Aria CV, AstraZeneca, Axon Therapies, Bayer, Boehringer Ingelheim, Boston Scientific, BridgeBio, Bristol Myers Squibb, Corvia, Cytokinetics, Edwards Lifesciences, Eidos, Imara, Impulse Dynamics, Intellia, Ionis, Eli Lilly, Merck, NGM Biopharmaceuticals, Novartis, Novo Nordisk, Pfizer, Prothena, Regeneron, Rivus, Sardocor, Shifamed, Tenax, Tenaya, and Ultromics. All other authors have reported that they have no relationships relevant to the contents of this paper to disclose.
